# Milk: From a Medico-Sanitary Standpoint*An essay read before the Keystone Veterinary Medical Association of Philadelphia.

**Published:** 1886-01

**Authors:** Thomas B. Rogers

**Affiliations:** Demonstrator of Anatomy Veterinary Department, University of Pennsylvania


### ART. III.—MILK: FROM A MEDICO-SANITARY STANDPOINT.*

BY THOMAS B. ROGERS, D.V.S.

*Demonstrator of Anatomy Veterinary Department, University of Pennsylvania.*

*Anatomy of the Mammary Gland of the Cow.*—The mammary gland in the cow is quadruplex, each quarter having its teat; behind these are usually found two rudimentary teats; these are rarely perforate, and more unfrequently still, give milk.† The blood supply of the gland is principally derived from branches of the external pudic artery, and is returned by the subcutaneous abdominal veins. These in milch cattle attain enormous volume and are commonly spoken of as the "milk veins;" the openings through which they enter the abdominal walls are termed the "milk fountains" or "milk doors."

The nerves are derived from the first lumbar pair, filaments from the sympathetic also reach the gland. The skin of the udder is thin, soft, devoid of pigment and sparsely covered with delicate, silky hair, always light in color. On the back part of the udder and on the inside of the posterior portion of the upper part of the thighs the hair looks upward and constitutes the "escutcheon." The size and shape of the escutcheon is held to be indicative of the milk value of the cow, and the different shapes and sizes have been tabulated and systematized by Guenon. It has been suggested that the escutcheon owes its origin to a dragging down of the roots of the

* An essay read before the Keystone Veterinary Medical Association of Philadelphia.

† The writer recently attended a cow whose supernumerary teats occupied each side of the lips of the vagina. Mr. E. S. Dobbs, of Mt. Ephraim, N. J., had a black cow that milked from six teats.

the hair by the heavy contents of the udder, in the same way that a bag made of horsehair would have the direction of the free ends of the hair reversed if filled by a heavy substance.

*Histology.*—The mammary glands are conglomerate, the lobules formed by the union of the alveoli, from these the ducti lacteferi, excretory ducts, empty into the galactiferous sinuses at the base of the teats. The alveoli have during lactation a clovate form, a length of 105-180 mm. and a breadth of 60-180 mm. They are lined by cylindrical epithelium with finely granular cell contents, round or oval nucleus, with nucleolus which is sometimes double. The inter-alveolar connective tissue is fine, fibrous, containing oval and spindle-shaped cells, often united to the membrana propria by their processes. Here, also, are lymph spaces communicating with the lymph capillaries. The lacteal ducts are lined by a single layer of cylindrical epithelium, from 15-18 mm. in length and 6-12 mm. in breadth. The arteries and veins follow the course of the excretory ducts, the capillaries wind spirally around the alveoli, and form a network upon the external surface of the membrana propria.

*The Secretion of Milk.*—The fat of milk is the result of the activity of certain cells of the gland. It may be seen to gather in them in the same way as in adipose tissue, and is thence ejected either by rupture of the cell wall, or by an extrusive process similar to that by which the amœba rejects the undigested food. That the fat is formed by the gland and not separated from the nutritive fluids is shown by the fact that its formation is quantitatively increased by a protein, and decreased by a fatty diet. A bitch fed on meat gave off in fat through the milk a greater amount than she received with her food, gaining in flesh the while, so the supply did not come from her own reserve of adipose tissue.

The casein and lactin are likewise products of the activity of the cells of the gland. The salts are probably partly separated from the blood, in part transformed by the cells: the water is separated from the nutritive fluids. The excretion is so far under control of the will that the cow can " hold up her milk," giving considerable trouble to the milkman, and the influence of the nervous system on the secretion is considerable. Sudden fright or excitement arrests the flow, while an easy life and pleasant surroundings increase and favor it both in women and cattle The flow is promoted by regularity in the hour of milking, and by letting the cows have food before them when they are milked. (Prof. Huidekoper informs me that in the Netherlands the cattle are milked three times daily, and that the yield per diem is thus considerably increased).

*Composition.*—Milk is a fluid of somewhat complex composition, containing, as it does, all classes of food stuffs—protein, fatty, saccharine, and saline in an aqueous menstruum, and while its qualitative composition differs but little, the quantitative offers considerable variation in the different species of animals.

From these data we learn that the milk from an average cow contains from 12 to 13 per cent. of solids, and from 3 to 6 per cent. of fat. A remarkable feature of the analyses is the constancy of the solids not fat; whether the milk is rich or poor in cream, the solids not fat vary little from 9.25 per cent.; indeed, it has been proposed to make this the most constant factor—the standard on which prosecutions for watering shall be based. The average of nine analyses by R. O. Doremus, made for the defense in the case of the "State of New York *v.* Schrumpf" gives solids not fat 8.32 per cent. I have gone over every attainable analysis in a considerable course of reading, and can find none where the solids not fat come down so low, except in a paper on the Holstein cow, by

TABLE OF COMPOSITION IN DIFFERENT SPECIES.

| Species. | Water. | Fat. | Casein. | Lactin. | Salts. | Total Solids | Authority. |
|---|---|---|---|---|---|---|---|
| Woman.... | 88.06 | 3.40 | 3.70 | 4.50 | .30 | 11.90 | Simon. (*a*) |
| Woman.. . | 88 90 | 2.66 | 3.92 | 4.36 | .13 | 11.07 | M. Foster (*b*) |
| Goat ...... | 84.49 | 5.68 | 3.51 | 3.69 | 61 | 13.49 | Smith. (*c*) |
| Ewe ...... | 83.23 | 5.13 | 6.97 | 3.69 | .71 | 16.50 | " |
| Ass....... | 89.00 | 1.85 | 3.56 | 5.05 | .54 | 11.00 | " |
| Mare...... | 90.43 | 2.43 | 3.33 | 3.27 | .52 | 9.55 | " |
| Sow .... . | 85.49 | 1.95 | 8.45 | 3.03 | 1.09 | 14.52 | Fleming. |
| Bitch.... .. | 77.20 | 8.79 | 11.68 | 1.59 | .78 | 22.84 | " |
| Camel..... | 86.60 | 3.60 | 4.00 | 5.80 | not giv'n | 13.40 | " |
| Llama .... | 86.50 | 3.10 | 3.90 | 5.60 | .80 | 13.90 | " |
| Cow.... .. | 86.00 | 3.60 | 5.50 | 3.80 | .66 | 13.56 | Smith. (*c*) |

(*a*) Frey Hist., p. 554. (*b*) Phys., p. 442. (*c*) On Foods, p. 315.

TABLE OF COMPOSITION OF COWS' MILK BY DIFFERENT OBSERVERS.

| Water | Fat. | Casein | Lactin | Salts. | Total Solids | Solids not Fat. | Authority. | Reference | Remarks. |
|---|---|---|---|---|---|---|---|---|---|
| 86.66 | 3.80 | 4.37 | 4.54 | .63 | 13.34 | 9.54 | Chandler. | State N.Y. *v* Schrumpf. | |
| 85.96 | 4.00 | 5.01 | 4.30 | .73 | 14.04 | 10.04 | Wanklyn. | Milk Analysis. | City milk. |
| 87.55 | 3.07 | 4.04 | 4.62 | .72 | 12.45 | 9.38 | " | " | Country milk. |
| 87.45 | 3.12 | 4.61 | 4.12 | .69 | 12.64 | 9.52 | " | " | Alderney milk. |
| 86.80 | 3.7 0 | 4.00 | 4.80 | .70 | 13.20 | 9.50 | Letheby | Parke's Hygiene. | |
| 86.00 | 3.60 | 5.50 | 3.80 | .66 | 13.56 | 9.96 | E.Smith | Foods, p. 313 | |
| 90.15 | 2.91 | Casein & S'g'r | 6.24 | .68 | 9.83 | 6.92 | R.O.Doremus. | State N.Y. *v* Schrumpf. | Lowest 9 analyses. |
| 81.06 | 9.47 | Casein & S'g'r | 8.37 | .83 | 19.67 | 10.20 | " | " | Highest 9 analyses. |
| 86.50 | 5.18 | | | | 14.50 | 8.32 | " | " | Average 9 analyses. |
| 84.55 | 6.44 | | | | 15.45 | 9.01 | Shippen Wallace | From herd milk. | Highest 14 analyses |
| 87.32 | 3.21 | | | | 12.68 | 9.47 | " | | Lowest 14 analyses. |
| 86.27 | 4.28 | | | | 13.73 | 9.37 | " | | Average 14 analyses. |

Mr. James Neilson, of New Brunswick, published in the report of the State Board of Agriculture of New Jersey for 1882. The analyses there given were made during the Paris Exposition, on milk given by cattle there exhibited. The solids not fat are stated to be 8.15 for Ayrshire ; Short Horn 8.19, and Holstein 7.80. Struck by the low percentage, I wrote to Mr. Neilson relative to the matter, and in reply he says : " The protein is undoubtedly low, as are the total solids, possibly owing to the feed and surroundings of the cattle. They were very much disturbed, owing to the tens of thousands of visitors daily."

From the constancy of the solids not fat we learn that the qualitative difference in milk from different breeds of cattle is principally in the fatty element, and I do not think that in any breed the casein is sufficiently in excess to warrant their selection for the cheese dairy on that ground: indeed, all goes to show that however much we may have increased the quantitative yield of milk by a forced survival of the fittest, extending over many generations, its qualitative composition is, save within very narrow limits, beyond our control. We cannot alter it radically any more than we can alter the composition of the blood. Could we do so, we could build up races of giants or pigmies at will.

*Milk Fat.*—The fat exists in milk in an emulsified state, and it is to the regular diffusion of this ingredient that the fluid owes its opacity. Its composition is:

| | | |
|---|---|---|
| Margarine | 68 | per cent. |
| Oleine | 30 | " |
| Fatty Flavoring Acids | 2 | " |

Its point of fusion is, commencing 65°-68°, completing 80°-90° F.

*The Casein* (C. 53.83; O. 22.52; H. 7.15; N. 15.65 per 100 parts), when freed from fat and in the moist condition, is a white, friable, opaque body; readily soluble in dilute acids or alkalies, and re-precipitated on neutralization. If, however, potassium phosphate is present, as in milk, the solution must be strongly acid before any precipitate is obtained. Casein, as occurring in milk, has had several reactions ascribed to it as characteristic, but these lose their importance on considering that milk contains in addition to casein other substances, such as potassium phosphate, and a number of bodies which yield acids by fermentation. The presence of potassium phosphate has an especial influence on the reaction of casein. In the entire absence of this salt, acetic acid in the smallest quantities, as also CO2, gives a precipitate, but if this salt is present CO2 gives no precipitate, and acetic acid only when the solution is acid from the presence of free acid, and not from that of acid potassium phosphate.

Besides casein milk contains some albumin not precipitated by acids, and if milk is kept at 85° F. out of the body, the casein is increased at the expense of the albumin.

When the action of the cell is imperfect, as at the beginning and end of lactation, the albumin is in excess of the casein; but when the cells are in full functional activity the formation of casein is prominent (Foster). In addition to casein and albumin, milk, according to Millon, contains another albuminoid substance in small amount, which he calls lacto-protein.

*The Milk Sugar* (C12 ; H24 ; O12) has a right rotating power of 93.1° for yellow light in aqueous solution; this power diminishes slowly on standing, rapidly if boiled, until it remains constant at x 53.3° : the amount of rotation is independent of the concentration of the solution. It crystalizes in hard, colorless, four or eight-sided prisms, soluble in six parts of cold and two parts of boiling water, entirely insoluble in alcohol or ether, fully precipated from its solutions by the addition of lead acetate and ammonia. Lactose may be said to admit of no direct alcoholic fermentation. This may, however, sometimes be induced by a lengthened action of yeast. By boiling with dilute mineral acids, lactose is converted into galactose, which readily undergoes the alcoholic fermentation. This body is readily soluble in water, and has a permanent rotatary power of x 83.2°. When milk is allowed to stand the lactose passes into lactic acid, and it must be remarked that while isolated lactose is incapable of direct alcoholic fermentation, milk itself may be fermented. Berthelot was unable in this direct alcoholic fermentation to detect any intermediate change of the lactose into another fermentable sugar (Foster).

*Milk Salts.*—

PERCENTAGE OF SALTS. (*a*).

| | |
|---|---|
| Calcium Phosphate | 0.3 |
| Magnesium " | .06 |
| Ferrum " | .007 |
| Potassium Chloride | .17 |
| Sodium " | .03 |
| Soda, free | .04 |
| | .607 |

———o———

IN 100 PARTS OF MILK SALTS THERE ARE (*b*)

| | |
|---|---|
| Potash | 23.46 |
| Soda | 6.96 |
| Lime | 17.34 |
| Magnesia | 2.20 |
| Chloride Potass | 14.18 |
| " Sodium | 4.74 |
| Phosphoric Acid | 28.40 |

(*a*) Smith, on Foods, p. 316 ; (*b*) op. cit., p. 317.

*Colostrum.*—The first milk secreted after delivery—the "colostrum," *anglice*, beaslings or beastings—differs considerably in composition from normal milk. Its specific gravity is increased, being in cows' milk 10.63; the free fat globules are smaller than normal, and are present in diminished quantity. There are also present numerous "colostrum corpuscles," larger than normal fat cells, of spherical or ovoid shape; they are usually found in agglomerated masses. Colostrum also contains active leucocytes. Fleming states that the colostrum corpuscles are epithelial cells, with fatty contents, and also states that albumin largely takes the place of casein in this, the first secretion of the gland. Toward the end of lactation, if the animal is pregnant, albumin again takes the place of casein, the milk coagulates with heat, and the sugar decreases in amount or disappears altogether. The colostrum has purgative properties, being apparently a device of nature for ridding the intestines of their contents accumulated during intra-uterine existence—the meconium. At or about the fifth or sixth day of lactation the colostrum is replaced by ordinary milk.

COMPOSITION OF COLUSTRUM.

| SPECIES. | Water | Fat. | Albumin | Lactin. | Mucus. | Authority. |
|---|---|---|---|---|---|---|
| Cow........ | 75.80 | 2.60 | 15.00 | 3.60 | Salts, 3.00 | Boussingault. |
| Cow........ | 80.33 | 2.60 | 15.07 | Traces. | 2.00 | Dumas. |
| Ass ....... | 82·84 | 5.60 | 11.60 | 4.30 | .70 | " |
| Goat....... | 64.10 | 5.20 | 24.50 | 3.20 | 3.00 | " |

COMPOSITION OF BUTTER-MILK AND SKIM MILK.

| | Water | Fat. | Casein. | Lactin. | Salts. | Authority. |
|---|---|---|---|---|---|---|
| Buttermilk.. | 88.00 | .70 | 4.10 | 3.60 | .80 | Smith. |
| Skim milk.. | 88.00 | 1.80 | 4.00 | 3.80 | .80 | " |

From the table of composition it will be seen that buttermilk approaches in composition to skim milk. It is a valuable article of diet, in health, especially for the poor, whose dietary is apt to be deficient in nitrogen. In many parts of the British Islands it constitutes an important article of food, and its use in India is universal. As a food it is regarded as so important by the pastoral tribes of Meerut that they say "a man may live without bread; without buttermilk he dies." There are about 336 grs. of carbon, and 35 grs. of nitrogen in a pint of buttermilk.*

*The Inspection of Milk.*—Owing to the fact that milk mixes in all proportions with water, the pump has been from time immemorial the milkman's best friend. Water is added in all proportions, from the lump of ice placed in the can without dishonest intentions, to the 60 per cent. of water and 40 per cent. of milk peddled by a dealer in New Jersey who, when caught, was selling 34 quarts off a yield of 14. How can this fraud be detected? In a limited degree it cannot be detected at all—we must grin and bear it.

With a view to limit this fraud, examinations have been made of a great many samples of milk, in order to determine its minimum specific gravity, and in mixed milk from healthy cattle this has not been found below 10.29 at 60° F.

Here the experimenters had something to work on, a hydrometer adapted to the fluid would do the work, hence the lactometer; this instrument, valuable though it is, cannot be said to have fulfilled the high hopes entertained for it. If a hydrometric reading of alcohol is taken at a temperature of 70° F., a reference to Piles' tables shows at once what the result represents at 60° F. This cannot be done with milk. It is not a fluid of definite chemical composition, and the expansibility of its components varies with alterations of temperature.

Thus a sample of milk tested by Prof. Chandler, which stood at 100° of the lactometer at 60° F., was found to stand at 106° at 44° F.; at 98° at 66° F.; at 90°, at 80° F., and at 74°, at 100° F.† Again, the fatty portion of milk is the lightest;

* Smith, on "Foods," p. 329.

† Mott, on "Milk," p. 23.

consequently the more fat the less the specific gravity, so, were we blindly reliant on the lactometer we might condemn as watered a milk rich in fat and all solids. Conversely, the removal of the cream increases the specific gravity, and a careless official might compliment a high-standing sample, while the milkman laughed in his sleeve, and drove round the corner to sell the cream he had removed. We have talked about the lactometer. What is it? It is a very delicate hydrometer, its scale running from 1000, marked 0 on the scale, to 1034, marked 120; that is to say, it has a range from a specific gravity of water to that of an extremely rich sample of whole milk, or thoroughly skimmed milk. I am speaking of the New York Board of Health instrument. The French demand a specific gravity of 1030. Many of the instruments of the shops are graduated carelessly, and are therefore worthless, and an inspector's instrument should be compared with a standard one before being used. It is curious to note how sanitary authorities differ in regard to the value of this instrument. The New York Board of Health prosecutes and gets convictions on its readings. Wanklyn, the English analyst, says it is utterly unreliable. In the State of New Jersey, a middle course is taken—the lactometer is used in examinations, but prosecutions are based on the percentage of solids as shown by chemical analysis. The instrument must be used along with a level head. The idea that milk so rich in cream as to send the lactometer below a hundred would be condemned is on the face of it absurd. In the first place, very few dealers handle milk of this description without letting it down prior to offering it for sale. In the second, a sample of this character would at once attract attention to its physical properties, appearance, taste, method of leaving the lactometer (milk rich in cream clings to the instrument, leaving it slowly; watered, and especially skimmed milk leaves it at once on withdrawal). On the other hand, if a sample goes above 110 without being manifestly full-bodied, it is fair to presume that a portion of the cream has been removed. The real difficulty with the lactometer is this: to allow for the production of milk poor in solids the standard is placed far too low for the bulk of the milk raised; thus, taking a sample of pure milk—S.G., 1.0314; lact., 108, we get the annexed result by watering the whole milk and the same sample skimmed.

| | Unskimmed. | | Skimmed. | |
|---|---|---|---|---|
| | S. G. | Lact. | S. G. | Lact.* |
| Pure Milk.................... | 1.0314 | 108 | 1.0337 | 117 |
| 10 per cent. Water............ | 1.0295 | 102 | 1.0308 | 106 |
| 20 " " ............ | 1.0257 | 88 | 1.0265 | 91 |
| 30 " " ........... | 1.0233 | 80 | 1,0248 | 86 |
| 40 " " ........... | 1.0190 | 66 | 1.0208 | 72 |
| 50 " " ............ | 1.0163 | 56 | 1.0175 | 60 |

It will be seen that 10 per cent. of water can be added before the 100 mark is reached, and to richer milk still more water could be added. The inspector will often be asked by dealers or producers why the standard is fixed at a certain percentage of solids, and he cannot answer the question better than to quote the words of Dr. W. K. Newton, State Inspector of New Jersey. "A word here as to the claim that the standard is an average. In answer to this I shall say that the standard is not *an average*, but a limit, and was not arrived at by averaging, but was adopted when it was conclusively shown that the milk produced in this State *never went below* 12 per cent. solids. Persons who object to the standard would usually resist the legalizing of any limit, and the objection is not so much against the limit fixed by law, as it is against any limit whatever.†

Good milk is creamy white or yellowish in tint, is thoroughly opaque, shows no blue line around the edge of the containing vessel, has a full, satisfying, somewhat unctuous taste, and (except its characteristic animal smell) is odorless ; it is feebly acid or neutral in reaction. (Fleming says it is acid in carnivora, alkaline in herbivora).

To examine milk with the lactometer the sample must be thoroughly stirred, to insure the admixture of the partially separated cream, a quantity removed into the hydrometer jar or tin, and the lactometer allowed to sink gently into it, taking

* Mott, on "Milk," p. 25.

† Dr. W. K. Newton, 7th Rep. N. J. S. B. H., 1883, p. 255.

care not to wet the stem above the 100 mark during its insertion, as the small amount of adherent fluid would make some difference in the reading.

The vessel should be full, in order to allow the reading to be correctly taken. After taking these precautions, should the sample go below 100 at 60° F. or below 60° a "serving kettle" should be procured, and a quantity of the milk poured repeatedly from one vessel to the other. It should then be tested again. This should always be done, in justice to the dealer, and I have seen it make a difference of several degrees. After this, if the lactometer still stands below 100 at a temperature of 60° F., or below 60°, a sample must be taken in a perfectly clean, dry bottle (four ounces is a sufficient quantity, and a number of clean dry corked four-ounce bottles should be carried in the inspector's valise for this purpose). The bottle must be sealed in the presence of a witness and should bear a label on which the name of the shipper, the dealer's name, their residences, the date, and the inspector's name should be plainly written. What is to be done with the condemned milk? In the earlier operations of the milk law in New Jersey it was thrown into the gutter, and the power still remains with the inspector to follow this course if he thinks proper. I have never done this. The mind of the public is apt to be shocked at the apparently useless waste of valuable property, and the same end can be attained by shipping the condemned milk back to the producer, "freight forward." It is only fit for the swill-barrel when it reaches its destination. In winter, when milk is frozen, or nearly frozen, in the cans, it is not practicable to use the lactometer, and at this season the inspector should confine himself to taking samples, two cents' worth of milk being purchased from each dealer, placed in a bottle, sealed, and submitted to the analyst. If he finds the specific gravity and general appear ance of the sample all right, there is then no need to analyze it.

Particular attention should be paid by the inspector to places where milk is sold by the glass—confectionery stores, restaurants, milk depots. In the State of New Jersey skimmed milk is not allowed to be offered for sale in cities of the first-class (Newark and Jersey City), the State Inspector finding it impossible to stop its substitution for whole milk by other means, though it is probable that the infliction of a heavy penalty (say $200, with imprisonment at the discretion of the court) would stop it. A small fine is useless. The dealer can pay it occasionally, and still make money out of the fraud.

Whatever the standard assumed, it must be rendered arbitrary, prosecution must as quickly follow a falling below the standard of a quarter of one per cent. as for a shamefully watered sample. The State Inspector of New Jersey has set all sanitary officers an example in this regard. A milkman pulled up for a sample containing total solids, 11.80 or 11.90, will meet with no better treatment than he would had he reduced the solids one-half. This is as it should be, and is the only way to enforce laws bearing on sanitary medicine; the "give an inch and take an ell" applies nowhere better than here. In works on sanitary medicine and food adulteration, a number of sophistications, besides watering and skimming, are mentioned; those mentioned in the English works are rarely used here, and those most frequently used in this country (milk preservatives) find no mention in any works I have consulted. Bicarbonate of soda is added to correct acidity; annatto, turmeric and yolk of egg, to give color and apparent richness; solutions of gelatine or isinglass, to give body; chalk, to increase the specific gravity and correct acidity. Sheep's brains are stated to have been found in milk. The preservatives in common use are boracic acid, borax, salicylic acid in small quantities, and the patented preservatives, "Rex Magnus" and "Humiston."

It is sometimes convenient to make a rough determination of the cream without resorting to analysis. This may be done in two ways:

*First.*—By taking a long test-tube, and pasting on it a slip of paper, divided into 100 equal parts by the compass; fill it with the milk, place it in a closet free from currents of air, let it remain twenty-four hours, and note the number of divisions on the slip which are occupied by the cream (Wanklyn states that 1 gramme cream = 0.2 gr. of fat). There is, however, considerable difference in the readiness with which different samples of milk "throw up their cream," and this, in a degree, renders this test valueless. Thus, it is asserted that a "butter cow," having the fat globules of her milk of larger size than a "cheese cow," throws up her cream more readily and completely, thus getting a reputation through the "creamometer" not sustained by analysis.

The *second* method is to examine with the "lactoscope." This

LACTOSCOPE TABLE.

| CC. Milk | To 100 parts of water obscures the light= | | | | | Per cent. Fat. | |
|---|---|---|---|---|---|---|---|
| 1. | " | " | " | " | " | 23.43 | |
| 1.5 | " | " | " | " | " | 15.69 | |
| 2.0 | " | " | " | " | " | 11.83 | |
| 2.5 | " | " | " | " | " | 9.51 | |
| 3.0 | " | " | " | " | " | 7.96 | |
| 3.5 | " | " | " | " | " | 6.86 | |
| 4.0 | " | " | " | " | " | 6.03 | |
| 4.5 | " | " | " | " | " | 5.38 | |
| 5.0 | " | " | " | " | " | 4.87 | |
| 5.5 | " | " | " | " | " | 4.45 | |
| 6.0 | " | " | " | " | " | 4.09 | |
| 6.5 | " | " | " | " | " | 3.80 | |
| 7.0 | " | " | " | " | " | 3.54 | |
| 7.5 | " | " | " | " | " | 3.32 | |
| 8.0 | " | " | " | " | " | 3.13 | |
| 8.5 | " | " | " | " | " | 2.96 | |
| 9.0 | " | " | " | " | " | 2.80 | |
| 9.5 | " | " | " | " | " | 2.67 | |
| 10.0 | " | " | " | " | " | 2.55 | |
| 11. | " | " | " | " | " | 2.43 | |
| 12. | " | " | " | " | " | 2.16 | |
| 13. | " | " | " | " | " | 2.01 | |
| 14. | " | " | " | " | " | 1.88 | |
| 15. | " | " | " | " | " | 1.78 | |
| 16. | " | " | " | " | " | 1.68 | |
| 17. | " | " | " | " | " | 1.60 | |
| 18. | " | " | " | " | " | 1.52 | |
| 19. | " | " | " | " | " | 1.45 | |
| 20. | " | " | " | " | " | 1.39 | |
| 22. | " | " | " | " | " | 1.28 | |
| 24. | " | " | " | " | " | 1.19 | |
| 26. | " | " | " | " | " | 1.12 | |
| 28. | " | " | " | " | " | 1.06 | |
| 30. | " | " | " | " | " | 1.00 | |
| 35. | " | " | " | " | " | .89 | |
| 40. | " | " | " | " | " | .81 | |
| 45. | " | " | " | " | " | .74 | |
| 50. | " | " | " | " | " | .69 | |
| 55. | " | " | " | " | " | .64 | |
| 60. | " | " | " | " | " | .61 | |
| 70. | " | " | " | " | " | .56 | |
| 80. | " | " | " | " | " | ·52 | |
| 90. | " | " | " | " | " | .49 | |
| 100. | " | " | " | " | " | .46 | |

instrument consists of a little cup, formed by two parallel pieces of glass, distant half a centimetre from each other, and closed everywhere except at the top. The auxiliary apparatus consists of a glass, graduated to 100 C C., and a pipette having a capacity of 5 C C. 100 C C. of water are placed in the measure, and 2-3 C C. of the milk to be tested are added; the "lactoscope" is then filled with this diluted milk, and a candle placed one metre distant from the apparatus is looked at through it. Should the contour of the flame be seen, the milk is poured back into the large measure and more undiluted milk added, this being repeated until the mixture obscures the contour of the flame; the percentage of fat is then calculated by the following formula, determined by a comparison of the results given by the lactoscope and by chemical analysis:

Let x = percentage of fat, and m the number of CC. of milk required.

Then $x = \frac{23.2}{m} + 0.23$.

Thus, if 3 CC of milk were required to obscure the light, the equation would stand

$$x = \frac{23.2}{3}\ 0.23 = 3\,x = 23.2 + 0.69 =$$

$$3\,x = 23.89 = x = 7.96 = \text{p. c. of fat in milk.}$$

To save the trouble of calculation, the preceding table has been made.

It now remains to examine the results obtained by this instrument. The following were obtained by Dr. Heeren:*

| No. | Chemical Analysis. | | Lactoscope. | | No. |
|---|---|---|---|---|---|
| 1 | Fat | 3.160 | Fat | 3.140 | 1 |
| 2 | " | 5.018 | " | 5.610 | 2 |
| 3 (*) | " | 4.225 | " | 5.000 | 3 |
| 4 (†) | " | 2.312 | " | 1.660 | 4 |

Now skimmed milk contains smaller fat globules than intact milk, and these cause a greater cloudiness in proportion to the existing quantity of fat than larger ones, and as the optical

* Mott, on "Milk," p. 49, *et seq.* † Nos. 3 and 4 somewhat skimmed.

test is based on the degree of opacity, its results are not equable for skimmed and whole milk or watered milk. In watered or whole milk the lactoscope demonstrates the fat with considerable exactness; in skimmed milk it favors the dealer. Its great value is to the producer, as he is thereby furnished with a ready means of ascertaining the percentage of fat at little trouble or expense. Indeed, by the aid of the lactoscope we may make a quasi-analysis of milk without any very delicate apparatus, by thoroughly drying 100 C C. until it ceases to lose weight we get the total solids. A lactoscopic examination gives the fat, and this subtracted from the total solids gives the solids not fat.

TABLE SHOWING VALUE OF THE DEGREES OF THE NEW YORK BOARD OF HEALTH LACTOMETER IN SPECIFIC GRAVITY.

(From Mott, on "Milk.")

| Lact. | S. G. | Lact. | S. G. | Lact. | S. G. | Lact. | S. G. |
|---|---|---|---|---|---|---|---|
| 0 | 1.00000 | 31 | 1.00889 | 61 | 1.01769 | 91 | 1 02639 |
| 1 | 1.00029 | 32 | 1.00928 | 62 | 1.01798 | 92 | 1.02668 |
| 2 | 1.00058 | 33 | 1.00957 | 63 | 1.01837 | 93 | 1.02697 |
| 3 | 1.00087 | 34 | 1.00986 | 64 | 1.01856 | 94 | 1.02726 |
| 4 | 1.00116 | 35 | 1.01015 | 65 | 1.01885 | 95 | 1.02755 |
| 5 | 1.00145 | 36 | 1.01044 | 66 | 1.01914 | 96 | 1.02784 |
| 6 | 1.00174 | 37 | 1.01073 | 67 | 1.01943 | 97 | 1.02813 |
| 7 | 1.00203 | 38 | 1.01102 | 68 | 1.01972 | 98 | 1.02842 |
| 8 | 1.00232 | 39 | 1.01131 | 69 | 1.02001 | 99 | 1.02871 |
| 9 | 1.00261 | 40 | 1.01160 | 70 | 1.02030 | 100 | 1.02900 |
| 10 | 1.00290 | 41 | ).01189 | 71 | 1.02059 | 101 | 1.02929 |
| 11 | 1.00319 | 42 | 1.01218 | 72 | 1.02088 | 102 | 1.02958 |
| 12 | 1.00348 | 43 | 1.01247 | 73 | 1.02117 | 103 | 1.02987 |
| 13 | 1.00377 | 44 | 1.01276 | 74 | 1.02146 | 104 | 1.03016 |
| 14 | 1.00406 | 45 | 1.01305 | 75 | 1.02175 | 105 | 1.03045 |
| 15 | 1.00435 | 46 | 1.01334 | 76 | 1.02204 | 106 | 1.03074 |
| 16 | 1.00464 | 47 | 1.01363 | 77 | 1.02233 | 107 | 1.03103 |
| 17 | 1.00493 | 48 | 1.01392 | 78 | 1.02262 | 108 | 1.03132 |
| 18 | 1.00522 | 49 | 1.01421 | 79 | 1.02291 | 109 | 1.03161 |
| 19 | 1.00551 | 50 | 1.01450 | 80 | 1.02320 | 110 | 1.03190 |
| 20 | 1.00580 | 51 | 1.01479 | 81 | 2.02349 | 111 | 1.03219 |
| 21 | 1.00609 | 52 | 1.01508 | 82 | 1.02378 | 112 | 1.03248 |
| 22 | 1.00638 | 53 | 1.01537 | 83 | 1.02407 | 113 | 1.03277 |
| 23 | 1.00667 | 54 | 1.01566 | 84 | 1.02436 | 114 | 1.03306 |
| 24 | 1.00696 | 55 | 1.01595 | 85 | 1.02465 | 115 | 1.03335 |
| 25 | 1.00725 | 56 | 1.01624 | 86 | 1.(2494 | 116 | 1.03364 |
| 26 | 1.00754 | 57 | 1.01653 | 87 | 1.02523 | 117 | 1.03393 |
| 27 | 1.00783 | 58 | 1.01682 | 88 | 1.02552 | 118 | 1.03422 |
| 28 | 1.00812 | 59 | 1.01711 | 89 | 1.02581 | 119 | 1.03451 |
| 29 | 1 00841 | 60 | 1.01740 | 90 | 1.02610 | 120 | 1.03480 |
| 30 | 1.00870 | | | | | | |

### MILK ANALYSIS.

*Total Solids.*—Evaporate 5 C C. on a water bath in a weighed platina dish until it ceases to lose weight. The evaporation must be continued for at least three hours. Multiply the result by 20 to get percentage. We thus at one stroke determine two factors—water and total solids.

*Fat.*—Treat the total solids with ether until they again cease to lose weight, weigh, note the loss and calculate percentage. Wanklyn directs that the fat be recovered from the ether and weighed, but as the ether extracts the fat only, the loss is represented by the fat, and the ether can be preserved for redistillation. Care should be taken that the ether is dry, or some sugar will be removed.

*Casein.*—Treat 10 C C. with acetic acid and boil, adding a considerable amount of water; let it stand twenty-four hours, pour off the supernatant fluid, dry the precipitate, treat with boiling ether to remove any adherent fat, dry again until the weight is constant, weigh and calculate percentage.

If a more accurate determination of casein is required the casein may be broken up into ammonia by permanganate of potash in the presence of an alkali and the albuminoid ammonia determined by Nessler's test. Each part by weight of casein gives by this process, 0.065 parts of ammonia, and the yield of albuminoid ammonia from 100 C C. of good milk is about 0.26 grammes. Full details for working the process will be found in works on water analysis.

*Lactin.*—Take a measured quantity of whey, evaporate to a constant weight on a water bath, and subject it to gentle ignition the residue subtracted from the total weight before ignition represents the lactin. Calculate percentage.

2*d Process.*—Take 10 C C. of milk, add a few drops of acetic acid, warm and filter; make up to 100 C C. with water, and put the filtered whey (which should be as clear as possible) into a burette. Place in a porcelain dish 10 C C. of Fehling's solution and add to it 20-30 C C. of water; boil. As soon as it boils briskly drop in the whey from the burette, taking care that the copper solution is left boiling. Continue the process until all the copper is reduced to sub-oxide and no blue color remains in the supernatant liquid, but stop before any yellow color appears. Read off the amount of whey used and divide by 10. The result is the amount of milk which exactly decomposes 10 C C. of the copper solution; this equals .0667 gr. of lactin; the amount of lactin in 10 C C. of milk is then found by proportion and the percentage determined by shifting the decimal point one figure to the right. Example, 15 C C. of diluted whey were required to reduce 10 C C. Fehling's solution representing 1.5 grammes original milk. Then

$.0667 \div 1.5 = 0.0445$ gr. lactin in 1 C C. of milk.
Therefore, $0.0445 \times 100 = 4.45 =$ per cent. of lactin.

*Ash.*—Incinerate dried solids, weigh, and calculate percentage. A very high per cent. of ash should be looked on with suspicion, as pointing to the addition of preservatives.

### ADULTERATIONS.

*Starch and Dextrin.*—The iodine test reveals the presence of starch, and with the addition of a drop or two of acetic acid and boiling, the presence of dextrin.

*Turmeric.*—A concentrated solution of the milk turns a deep brown when treated with alkalies: the cells may be detected by the microscope if the powder has been used.

*Annatto.*—The concentrated milk is orange red, and the color can be extracted by alcohol; it is turned purplish by acids and brighter red by alkalies.

*Soda Bicarb.*—The ash effervesces with acids, prove presence of base by blow-pipe test.

*Chalk* gives sediment and ash effervescent with acids.

### MILK AS A FOOD.

At what remote period the milk of animals was first used as a food by man we do not know, though its use is probably coincident with the time when men took the first step in civilization by the adoption of a pastoral life.

"The peasantry of Sweden and Norway, of Switzerland and the Tyrol, the Bedouins of Arabia, the people of Kurdistan and other mountainous countries in the East live in great part on milk." "Camels' milk is in common use among the Arabs and is often mixed with flour into a paste and boiled, when it is called Ayesh."

"It is also eaten largely by those living on the Thull, a sandy and unproductive district at Lera, in India, and it is stated to have a brackish flavor and little fat."* In the Old Testament allusions to milk are frequent. though apparently the milk of goats and camels was more used than that of the cow. So long as the tribes continued in a pastoral condition this may be accounted for by the greater readiness with which these animals moved from place to place, the milk supply being subordinate to their value as beasts of burden, or the value of their flesh as food. "And he took butter and milk and the calf which he had dressed, and set it before them." (Genesis xviii. 8.) Jacob gave Esau thirty milch camels and their colts. (Genesis xxxii. 15) "And thou shalt have goats' milk enough for thy food, for the food of thy household, and for the maintenance of thy handmaidens." (Proverbs xxvii. 27.) "Surely the churming of milk bringeth forth butter." (Prov. xxx. 33.) The milk of the buffalo is used in Egypt and is said by travellers to be of very rank and disagreeable flavor. During the first months of life milk constitutes the sole food of the mammalia, the young animal being a parasite of the mother. As shown by the table of composition it differs in composition in the different species of animals—the milk of the carnivora being the richest and that of the soliped herbivora the poorest in solids : indeed the milk of the bitch is so rich that great difficulty is experienced in bringing up puppies by hand, and in some parts of France where wet nurses suckle puppies in order to prolong lactation, the animals usually become rachitic. Milk also differs in composition at different periods of lactation. †

In the human species the amylolytic properties of the salivary and pancreatic glands are not developed until about the eighth month of life ; and starchy food should therefore be

---

* Smith on Foods, p. 318.

† Ewes' Milk, 3 weeks after lambing. Ewes' Milk, 6 weeks after lambing.

| | 3 weeks | | 6 weeks |
|---|---|---|---|
| Water, | 75.00 | Water, | 86.70 |
| Fat, | 12.78 | Fat, | 3.67 |
| Casein, | 6.58 | Casein, | 4.44 |
| Lactin, | 4 66 | Lactin, | 4.00 |
| Ash, | .98 | Ash, | 1.19 |
| | 100.00 | | 100.00 |

Fleming, Obstetrics, p. 255

withheld until this period is past. In the lower animals the development of the deciduous teeth and the instinctive weaning process adopted by the mother mark the time when the ability to grind, and digest food of its own seeking is attained by the young animal. In woman, and in the lower animals when in the wild state, the secretion of milk ceases when the young are weaned, but in the cow (and in some countries, the mare, ass, reindeer, sheep, camel and goat) the secretion is made permanent by the influence of domestication and selection—the cow often milking up to the time of the next parturition, and if the animal is spayed when in full milk the secretion may be continued for several years, the fat and casein being increased.*

When the mother cannot nurse her infant, or when there is reason that she should not do so, it is usual in this country to substitute the milk of the cow. (On the continent of Europe and in Great Britain the milk of the ass or goat is often used, and in 1819 Dr. Twierlien, a German, wrote a book recommending goats as wet nurses.) Asses' milk when attainable is probably the best, assimilating more closely in composition to mothers' milk than does that of the cow. During the first few months of life the milk should be diluted with an equal bulk of water, the amount of milk being gradually increased as the stomach grows better able to digest it, so that when the infant reaches about the ninth month, the amount of added water should be about one third; and when the first year is attained the milk may be given undiluted. The milk of one cow is usually preferred, though if the digestive organs are strong the infant will do equally well on herd milk.

Care should be taken that the source of supply is healthy well nourished, and amid good hygienic surroundings; should the milk disagree these points should at once be looked into. It often happens that while the milk agrees well with the child while the cow is "fresh," as time goes on, and the animal becomes again advanced in pregnancy, the increment of weight of the child is insufficient and dentition is tardy or arrested: under these circumstances a change of supply is imperative. If this cannot be done extra cream and limewater may be added to the milk.

---

* C. Schmidt, Amer. Vet. Rev., vol. 1, p. 316.

The cow should be milked at regular hours, as the retention of the milk in the gland for an hour or two after the regular time of milking will cause it to sour several hours sooner. Slop fed milk must be avoided, though if grains are fed moderately in conjunction with other food, the nutritive qualities of the milk do not seem to be impaired. Cattle pastured in meadows where access is had to aquatic plants are not a proper source of supply, the common spatter dock appears to be especially obnoxious in this regard. Milk sugar is better than cane sugar to give sweetness to the child's milk. The milk should be given at a temperature of 95° F. When the period of infancy has passed milk should still constitute a large portion of the child's food, indeed through the whole period of life milk must be regarded as a very important article of diet; and the suggestion that the aged may by its use smooth the way to the Euthanasia dates from the days of Galen.

*The Therapeutic Uses of Milk*—.Milk is given in disease because it presents in an easily assimilated form a considerable amount of nutrition, taxing the digestive apparatus but little, and offering no mechanical irritation to the alimentary tract. It is also used as a direct therapeutic agent. In acute diseases anorexia usually accompanies the fever, and when their course is prolonged the excessive oxidation of tissue leads to a degree of debility often overshadowing in importance the cause thereof: Thus in enteric fever it is not uncommon to see the fever break and leave the patient too exhausted to rally: the patient has worn out the disease, to be in turn worn out by the attendant debility, and the feeble spark goes out. So long as he can take and assimilate food one great source of anxiety is removed for the practitioner, and milk to-day constitutes the regimen of most of these cases, given with limewater or with alcoholic stimulants.

Should the stools contain undigested casein, recourse may be had to artificial digestion of the milk, and it may be proper in this connection to consider this question of artificial digestion. When milk reaches the stomach the casein is precipitated by the acid gastric juice, and the change from insoluble albuminoid to soluble peptone commences, then as the process continues it becomes, so to speak, retarded by its own activity; the envelopes of the fat cells are dissolved and the fat mixing intimately with the material undergoing digestion so sheathes the casein as to protect it measurably from the action of the gastric juice, and it is principally in the small intestine that milk is digested: the digestion is pancreatic and alkaline, not peptic and acid in character.

Now if in fever the stomach and pancreas have struck work, the chance for the digestion of milk are small indeed, and "an acute attack of intercurrent indigestion is often a veritable thundercloud in a clear sky to the practitioner; he must drop everything else and devote himself to tranquilizing the iritable organs of digestion.*

The digestion of the food by means of the pancreatic secretion as now offered by several chemists often enables him to do this and save life. When 5 grs. ext. pancreatic (Fairchilds) and 15 grs. of bicarbonate of soda are dissolved in a gill of water and added to a pint of milk, placed in a bottle and this immersed in a pitcher of hot water covered so the milk will retain its acquired heat, and allowed to stand for an hour and a half in a warm place artificial digestion of the milk takes place, the kypsin converts the albuminoids into peptones, the pancreatin emulsifies the fat, mechanically fitting it for absorption by the intestinal villi; the contents of the bottle become grayish-yellow in color, acquire a bitter taste, and the milk becomes perceptibly thinner: the result is milk ready for absorption. As great heat or cold stops the process, all that is necessary when the required degree of digestion is reached (to be determined by the condition of the patient's digestive organs), is to place the milk on ice or in boiling water; it may then be kept like ordinary milk. The added alkali serves a double purpose, it hastens the process and guards the product through the stomach. So active is this agent that if you add it to a bowl of bread and milk the milk will become thinner and the bread much softened during its leisurely consumption.

As a therapeutic hint it may not be out of place to remark that pancreatin, not pepsin, is the proper addition to nutritive enemata, the secretion of the rectum is alkaline not acid. Milk is a food of especial value where mechanical conditions (carci-

---

* Dr. Milner Fothergill. Indigestion and Biliousness.

noma of the stomach, gastric ulcer, irritation from poisons, stenosis of the pylorus, &c.), offer an obstacle to digestion by the pain they excite, or their interference with the passage of food along the canal. "When it becomes necessary to confine a patient for some time to a diet consisting exclusively of milk, great difficulty is often experienced in overcoming the repugnance of the patient. Although as a rule it is taken readily at first, after a time it begins to pall on the appetite and the greatest resolution is necessary on the part of the patient in order to continue it. A distressing sense of emptiness is experienced in the epigastrium. The mouth becomes pasty, and the tongue is coated with a thick whitish fur. Constipation, sometimes exceedingly obstinate, occurs, and the stools are hard and of an ochre yellow color. Occasionally diarrhæa is produced, but this is due to the fact that the milk disagrees and is not digested. The urinary secretion is increased in amount, but this is due simply to an increased flow of water. Although milk contains all the constituents necessary for the nutrition of the body, when it is used as an exclusive article of diet in the case of those accustomed to a full mixed diet, a decided diminution in the weight of the body takes place. After a time, however, the waste ceases, and the weight continues at a uniform level. The interference of a milk diet with nutrition is more decided when skimmed milk is used — a form in which it is usually administered in intestinal disorders. The pulse is quickened and the arterial tension lowered, but a fall in the pulse rate occurs when the body ceases to lose weight. A marked degree of debility is experienced by some persons, so that they are unable to take exercise. In two cases in which I used this treatment with signal success— chronic eczema, and chronic ulcer of the stomach—the patients, both females, experienced vertigo and faintness; and Mitchell mentions a case in which from the same cause he was obliged to discontinue the milk. Ordinarily, however, nothing more than weakness is experienced." BARTHOLOW.

With regard to the dose, Bartholow recommends the administration of 4 oz. every 3 hours at the commencement of the treatment, increasing the amount as the patient becomes able to bear it until he takes all he can assimilate.

Mitchell's limit is determined by the patient ceasing to lose weight. He uses milk only, for three weeks, making gradual additions to the diet, until about the sixth week the patient returns to a mixed diet, which for some months consists largely of milk. Dyspepsia, gastric catarrh, gastralgia, chronic diarrhæa, dysentery, ascites, anasarca, albuminuria, diabetes, eczema, gout, and gouty affections, have been removed by a persistence in this plan of diet. Owing to its diuretic properties milk is especially indicated in scarlet fever, reducing the danger arising from the attendant albuminuria.

Buttermilk is a useful remedy for the nausea of chronic alcoholism, and is a well known domestic application for tan or sunburn.

Formula for diet, drinks, &c., consisting principally of milk:

### Wine Whey.

Take 2 pints new milk, 4 oz. sherry wine. Heat the milk over a clear fire until nearly boiling, then add the sherry, simmer a quarter of an hour, skimming off the curd as it arises, then add a tablespoonful more sherry, and skim for a few minutes.—Dr. Bartholow.

### Arrowroot with Milk

Put into a saucepan to boil one pint of milk; stir very smoothly into a cup of cold milk a dessert spoonful of arrowroot. When the milk boils stir in the arrowroot, continuing to stir until it is cooked (5–10 minutes); then remove from the fire and sweeten to the taste.—Parrish.

### Egg Nogg.

Scald a tumblerful of new milk, not allowing it to boil; when cold add to it an egg, beaten to a froth, with sugar and a dessert spoonful of brandy.—Dr. Bartholow.

### Tapioca with Milk.

Tapioca two tablespoonfuls, water two teacupfuls, soak over night, add a little salt and a pint of milk, simmer until quite soft, stir well and while cooling pour into a bowl, sweeten to taste and add wine and nutmeg if desired.—Parrish.

### Castillon's Powders.

Powdered Tragacanth,
" Sago.
" Salep.
" Sugar, of each 1 oz.

Prepared oyster shells 2 drachms. Mix. Divide in powders weighing 1 drachm. Mix a powder with four tablespoonfuls of cold milk in a bowl, transfer to a milk pan, add gradually while stirring a pint of boiling milk, boil for 15 minutes and sweeten to taste.—Parrish.

### Peptonized Milk Gruel.

Take of good thick gruel, and cold milk each half a pint (Imperial), add the milk to the gruel while the latter is still boiling (the mixture will have a temperature of about 125° F.) To this mixture add 15 grs. ext. pancreatis (Fairchilds) and one scruple bicarbonate of soda, keep warm in a covered jug for two hours, boil and strain.—Adopted from Dr. Roberts.

### KOUMISS.

Milk may, by undergoing alcoholic fermentation, pass into a sort of milk wine, and following out the inborn desire of mankind for alcoholic stimulants, the nomad races of the Steppes convert the milk of the mare into "koumiss." Not alone, however, as a beverage is this used. It is stated, and that by some very competent authorities, to be a remedy of great value in phthisis and other wasting diseases. It is retained and assimilated when other food is rejected, and the increase of weight in some cases under its use borders on the marvellous. (Jagrelsky says that he has had patients gain as much as ten pounds a month, when no other food was taken). "Koumiss is prepared from milk by the addition of a ferment—some koumiss obtained from a previous fermentation, or dried koumiss—it is allowed to ferment three days at a temperature of from 70° to 80° F. It is then a bluish-white liquid, having a sharp, acidulous taste, and none of the characteristics of ordinary milk. If heated to 100° F. fermentation is definitely arrested. Allowed to stand after three days' fermentation it separates into three layers—the inferior caseous, the middle (an acid water), and the uppermost (a whitish fluid), the best koumiss." "The quantity of koumiss administered depends on the condition of the patient. In cases of feeble digestion (this being the only article of food) an ounce every hour will be a sufficient quantity. With increased facility in its digestion and assimilation, from a quart to a gallon per day may be taken. When it is used in connection with other food a tumblerful may be administered after each meal. It is estimated that each quart of koumiss contains four ounces of solid food. The tolerance to koumiss is remarkable, even in cases of gastralgia. It improves the appetite and excites the action of the kidneys. The patient experiences a pleasing exhilaration, due probably to the combined action of the alcohol and $C\ 0_2$°.

It favors sleep at night without leaving any after-headache." *

(The writer would, with diffidence, offer a remark here with regard to the diuretic properties of milk and some milk products. The diuresis is not, in his opinion, due to a drainage from the kidneys of an excess of fluid, but to a special diuretic action of milk sugar. He has noticed this increased flow of urine in himself and in his children after the administration of the milk sugar). Wanklyn gives the following analysis of koumiss : †

FULL KOUMISS.

| | |
|---|---|
| Water | 87.32 |
| Alcohol | 1.00 |
| C $0_2$ | 0.90 |
| Solids | 10.78 |
| | 100.00 |

THE SOLIDS CONTAINED

| | |
|---|---|
| Casein | 2.84 |
| Lactose and Lactic A | 6.60 |
| Fat | 0.68 |
| Ash | .66 |
| | 10.78 |

This analysis was made when the koumiss was 48 hours old.

SAME KOUMISS AFTER 6th DAY.

| | |
|---|---|
| Water | 88.47 |
| Alcohol | 1.60 |
| C $0_2$ | 1.50 |
| Solids | 8.43 |
| | 100.00 |

SAME KOUMISS AFTER 35th DAY.

| | |
|---|---|
| Water | 89.16 |
| Alcohol | 1.80 |
| C $0_2$ | 1.50 |
| Solids | 7.54 |
| | 100.00 |

SOLIDS NOW CONSISTED OF

| | |
|---|---|
| Casein | 2.57 |
| Lactose and Lac. A | 3.82 |
| Fat | .50 |
| Ash | .65 |
| | 7.54 |

MILK AS A CAUSE OF DISEASE.

Milk may communicate disease to man in several ways : It may undergo alterations, fermentative in character, through the presence of lowly forms of life, and thus acquire poisonous

* Bartholow, "Mat. Med. & Ther." pp. 24, 25.

† "Milk Analysis," p. 64, *et seq.*

properties; it may be the vehicle by which vegetable or mineral poisons are introduced to the human stomach after having first, so to speak, been filtered through the cow. It may carry to mankind germs of certain diseases having origin in the lower animals; and lastly, it may, after being drawn from the body, act as a carrier, and possibly as a culture liquid, for diseases affecting only the human species. It is important that we should bear in mind that the death rate does not depend so much on diseases well known in character, whose origin can easily be traced, as on the pernicious lowering of the vitality of the mass of the people through causes ill ascertained or obscure. It is not sufficient to say that the milk from a fevered animal has not been proven directly noxious; it cannot be normal milk, therefore its influence on health, though slight, is on the wrong side, and its use should be forbidden. Boards of health should take pains to keep themselves informed as to the sanitary condition of the cattle furnishing the milk supply in their jurisdiction; and the use of milk from diseased cattle, or of milk from farms where scarlet fever, enteric fever, measles, diphtheria, or other contagious diseases are present, should be forbidden until it can be shown that only the milk from sound cattle is utilized, or that there is no danger of the supply being contaminated by disease germs or excreta from sick persons.

One good step in this direction would be to prohibit the keeping of dairies within the limits or in the immediate suburbs of cities. Here, crowded for room, with insufficient drainage, ventilation and sunlight, it is impossible to keep healthy cattle. Dr. Miller, of Camden, and the writer, found many cows in stables of this description giving plenty of milk and eating well, but with accelerated respiration and temperature of 103° F. These cattle, when sick, are milked as long as milk can be drawn from the udder, are stuffed with grains (and those often in a state of decomposition) to increase the yield, and it is more by good luck than good management if the water supply is not contaminated by human or animal excreta; and it is rare that some water does not find its way into the milk; and as the limited space afforded by city and suburban lots ensures the milk being kept within the walls of the dwelling, the chances of contamination are great indeed.

The owners generally come from a dirty and ignorant element of our foreign population, to whom cleanliness represents the unattainable. If any one thinks this picture overdrawn, let them visit some of these places and judge for themselves.* So long as these city dairies cannot be suppressed every obstacle should be thrown in the way of their establishment. They should be registered and licensed, should be compelled to report promptly all cases of disease in their cattle or their family, and every infraction of the sanitary code promptly punished by fine, publication or revocation of the license.

### DISEASED MILK.

*Blue Milk.*—Two distinct varieties of blue milk have been observed ; in one, the milk is blue when drawn from the cow, it is of low specific gravity and throws up but little cream, and the blue tint can be distinctly seen through the cream after it has risen.

This variety of blue milk is not injurious to health, and is supposed to be due to cattle eating certain plants, as mercurius perennis, myosotis palustris, polygonum, aviculare, etc. The second variety of blue milk is fungoid in origin, and is in some parts of Europe a veritable scourge to the dairyman. In Normandy, Picardy, Artois, Holstein and the Marches of Brandenburg it has assailed the milk product over wide stretches of country. The milk appears healthy when first drawn from the cow, and the period antecedent to the first change has varied from eight to forty-two hours ; the cream becomes speckled with circular points and patches of an indigo-blue color, these gradually extending on and beneath the cream until the milk is uniformly blue. Hot weather is necessary to the development of this condition, and it ceases with the first frost. Kept long enough, the milk becomes covered with a rough white layer of the fungus, the blue becoming grayish blue, then gray, and finally of a dirty white hue, the cream becoming frothy from the evolution of gas. The whole of the milk in a

* The writer was called to attend a patient where, on a lot 20x80 feet, were congregated seven human beings, one horse, one cow, three pigs, one goat, a number of ducks, geese, fowls and rabbits, and several dogs and cats.

dairy is not at first attacked, but as time goes on the condition becomes general, and as the fungus may be carried in the air, one farm after another may become affected by it. Infusoria and monads are rapidly developed in the milk, and are most numerous in the blue patches. The reaction of the coagulated milk is less acid than usual, the casein is charged with the coloring matter, and is less consistent than in healthy milk. The color may be communicated to the whey but not to the butter.

*Causation.*—Braconnet attributed it to the cryptogam byssus cœruleus, Ehrenberg to the vibrio cyanogenus, later authorities to the oidium lactis, or "penicillium." Furstenberg has kept the fungus for more than a year without loss of its activity, its addition to milk at the end of that period being promptly followed by its reproduction. Haubner states that the coloration is confined to the albuminous envelop of the fat cells. If the milk be rendered alkaline the blue color is replaced by red or yellow, the after addition of acid restoring the original color. The influence of food on the change is not clear, though it is said that soil-covered, or mouldy forage favors its development, green clover, lupulin and lucern have been blamed for it, also the use of green forage after the dry food of winter, though from our knowledge of its fungoid origin, it would seem that these could only operate as the carriers of the proper cause.

It is also stated that congestion of, or hæmorrhage, into the mammary gland favors its development.

*Therapy.*—Change of pasture, cleanliness, disinfection of the dairy utensils (washing the pans, pails, hands, teats, etc., with weak chlorine water is recommended). The milk is of disagreeable taste, and gastritis and diarrhœa have followed its use. According to Grawitz, oidium lactis passes through the following stages of development: The conidia or spores, which are oval cells, lengthen out into one or more germinal tubes, which very soon become jointed and throw out lateral branches; these branches are at first cylindrical, but later become rounded off and appear as lengthened ovoids. Sooner or later these break up by transverse subdivision into chains or chaplets of conidia. The several conidia cells then recommence the developmental cycle, which proceeds as before. No true sporangia or spore capsules are formed, and no kind of sexual reproduction has been observed.*

*Red Milk.*—This, like the blue milk, may have a dual causation, *i.e.*, entrance into the milk of a coloring substance from the vegetation, or the presence of a fungus. Parmentier produced the first variety by feeding madder; when thus produced, it is probably innocuous, although its appearance would interfere with its sale. The second variety shows itself by the development of red patches in the layer of cream. It is usually seen in dirty dairies in bad weather, and its use has given rise to dyspepsia, choleraic attacks, and (in children) apthous affection.

*Yellow Milk* has also been observed, and oftentimes the yellow patches precede the formation of the blue. It has been attributed to the vibrio xanthogenus.

*Green Milk* is probably due to a mixture of the blue and yellow vibrios. The constituents of the bile and urine have been found in milk (due to faulty action of the liver on albuminoids ?) and milk from cows affected with phthisis is said to be calcareous.

### ABNORMAL FLAVOR.

*Bitter or Rotten Milk.*—This has a bitter, mawkish taste, and most disagreeable odor ; it is very difficult to make butter from it. The condition is probably due to bad housing, keeping, or adulteration with water containing decomposing animal matter.

The writer found a most execrable fecal odor in milk shipped in new cans; it was said by the dealer, a milkman of experience, to be due to the ingredients used in soldering the cans. It must not be forgotten in this connection that milk readily absorbs any odorous particles coming in contact with it, and every care should be taken to keep the milk house or cellar sweet and clean.

*Sweet Bitter Milk.*—This taste may be given to milk by the cattle feeding on vine or chestnut leaves; but in the form we are about to consider, the peculiar taste is not observed until the cream begins to rise. Fermentation proceeds in an irregular manner, and the cream has in places a yellowish color,

* Ziegler's "Pathology." W. Wood & Co., p. 318.

not unlike that of soup made from split peas; the rest of the cream may be natural looking, though dirty. The cream is frothy, and drops of oil appear on the surface of the milk (due to rupture of the fat cells or disintegration of their envelopes). The milk tastes remarkably sweet, this being succeeded by a bitter after-taste; the same peculiarity of taste is noticed in the casein. If the milk is kept for some time the taste becomes rancid, from splitting up of the fat; the odor is now disagreeable, and the milk soon becomes putrid.

It is very difficult to make butter from it, and if any is obtained it is tenacious, and resembles mucus of objectionable flavor, and quickly grows rancid; indeed, both butter and cheese made from this milk are uneatable. In inflammation of the udder blood or pus may find its way into the milk, and the same thing may happen when cattle are inoculated for contagious pleuro-pneumonia, blood or pus from the tail dropping into the pail. The red color due to blood may be recognized by the microscope, or the spectrum of hæmoglobine may be recognized if the cells are ruptured. Milk of this description is unfit for food. Taylor was consulted in a case where bloody milk caused vomiting and other symptoms of irritant poisoning in three children.* (The description of diseased milk is mainly condensed from Fleming's "Manual of Veterinary Sanitary Science and Police," to which the reader is referred if a fuller account is desired).

### MILK AS A VEHICLE FOR POISON.

Many poisonous and medicinal substances are excreted by the mammary glands, and milk may in this way convey them to persons or animals partaking of it. The custom of purging the infant through the milk of the mother is too well known to need comment. Gentian, aloes, and other nauseous drugs impart not only their taste, but their medicinal properties to the milk of the animal taking them; and the veterinarian must exercise discretion as to whether it is permissible to use milk from cattle when they are undergoing medicinal treatment. It is fabled that the habit of intoxication to which the Emperor Nero so immoderately yielded was owing to the influence thus

* Taylor on "Poisons," 3d Am. ed., p. 515.

exerted by a drunken nurse.* M. Jacquemin examined the milk of a cow which had been severely wounded at pasture; the wound had been dressed with carbolic acid, and he detected carbolic acid in milk drawn from the cow.† In a cow suffering from lead poisoning Taylor found traces of lead in the milk a few hours after the poison had been swallowed. ‡

Milk may also become impregnated with lead through being kept in badly-glazed pans. "A young woman who was taking tartar emetic for pleurisy suckled her infant, and it was observed that the child was attacked by a fit of vomiting after every attempt to suck the breast." § The case given by Dr. Fayrer ("Thanatophidia of India," p. 43) of a child dying from serpent poisoning taken in with the milk from its mother is in opposition to what is known relative to the action of this poison when taken into the stomach, and is probably to be explained by the existence of abrasions on the lips or in the mouth of the infant. Guenther found antimony in milk after feeding the tartrate to a cow. Harms observed a hæmorrhagic diarrhœa in two dogs and three young goats after feeding them with the milk of a cow which had been given forty-six grains of tartar emetic the day before. Klink demonstrated the presence of quicksilver in the milk of a woman afflicted with syphillis after inunction of mercurial ointment. A large number of persons in Rome were poisoned by the use of goats' milk; the disease, as it appeared in these people, was strongly characteristic of cholera. The goats were healthy, but had been fed on conium maculatum, clematis vitalba, colchicum autumnalis, and plumbago europea. Some of the vomited milk showed the presence of colchicum. ‖ The writer saw a valuable Jersey calf poisoned through drinking milk from an old paint keg.

As cattle have a habit of picking up and swallowing nails, bits of metal, paint skins, etc., care should be taken that they are not pastured where they can get at them, as in the neighborhood of rifle butts or chemical works.

* Dewees on Children. 11th Ed., p. 165.
† Taylor on Poisons. 3d Am. Ed., p. 55.
‡ Taylor on Poisons. 3d Am. Ed., p. 55.
§ Christison on Poisons. p. 483.
‖ Billings' Relation of Animal Diseases to the Public Health. p. 61.

*The Influence of Mental Emotion on the Secretion of Milk.*—Instances are on record which show that mental emotion may so modify the mother's milk that it becomes dangerous to the health or life of her progeny, and the nursing child will often accurately reflect the shades of the mother's temper. A child was attacked by convulsions after being suckled by a nurse who was at the time suffering from a fit of anger.* Sir Astley Cooper observed two cases where great mental emotion was followed by arrest of the secretion. Carpenter relates the case of a bitch whose puppy was seized with convulsions after sucking the enraged mother. Dr. Milner Fothergill quotes from Carpenter † the case of a woman who interfered in a quarrel between her husband and a soldier who was billeted on them. She snatched away the man's sword, broke it, and taking up her healthy infant, who was sleeping in the cradle, gave it the breast. In a few minutes the infant left off sucking, panted and fell back dead on the mother's breast. It is clear, says Carpenter, that the disordered secretion produced some very active poison, acting on the nervous system. Dewees gives the case of a child almost moribund from infantile diarrhœa, no amelioration was obtained until the mother, who had suffered from toothache, had the offending tooth removed. The child then recovered.‡

### DISEASES OF THE LOWER ANIMALS RENDERING THEIR MILK INJURIOUS TO HEALTH.

*Anthrax.*—Goheir has seen milk from cattle affected with anthrax, cause anthrax, on more than one occasion. Chisholm saw a case of anthrax in a three years' old child, caused by drinking the milk from an affected cow, and it has, in like manner, been conveyed to the lower animals.§

*Actinomycosis.*—It is entirely within the range of probability that actinomycosis has been conveyed to man by means of milk containing spores of the disease, and the fact that of the sixteen cases tabulated by Johne three had local lesions in the

---

* Evanson & Maunsell on Children. 2d Ed., p. 39.

† Indigestion and Biliousness. p. 99.

‡ Dewees on Children. 11th Ed., p. 275.

§ Fleming, Manual of Vet. Sanitary Science and Police. Vol. 2, p. 20.

mouth points very strongly in this direction.* (Actinomycosis, gr. *actis* a ray, *mycos* a fungus)—a disease most common in cattle, transmissible to other animals and to man, caused by the presence of a vegetable parasite, recognizable under the microscope by the ray-like arrangement of its spores. Its most common seat is on the jaw of the ox or cow, or in the substance of the tongue, where it occurs in yellow masses looking not unlike inflammatory products which have undergone caseation.

*Rabies.*—Authorities differ as to the possibility of transmitting rabies through the milk. Dolan says † "Milk may be used until symptoms of disease are manifested, may be given to animals, and owners may use it. People have remained healthy even after using milk at the commencement of the disease, but our researches prove that both flesh and milk are dangerous after the malady has developed."

Mr. Steele states ‡ "Two ewes were bitten by a rabid dog, rabies appeared in them about six weeks afterwards, and they were killed. One had two lambs, the other one; at first these lambs were allowed to suckle. They were subsequently attacked by rabies and were killed. It appears highly probable that they received the poison through the milk because they were removed from the ewes a month before they (the lambs) became affected, and there was no proof that they had been bitten." Billings says § that "the consumption of flesh and milk from diseased animals is not harmful," but as the immunity would probably depend on the possession of an intact mucous membrane along the alimentary tract, it would seem best to avoid it.

*Foot and Mouth Disease.*—The transmission of eczema epizootica to man through the medium of the milk has been placed beyond doubt by the experiments of Hertwig. "He drank daily for four consecutive days a quart of milk from cows having the disease. On the second day he observed a mild fever, pains in the limbs, headache, a dry and hot throat,

---

* Fleming. Actinomycosis. p. 35.

† Rabies, or Hydrophobia. p. 218.

‡ Taylor on Poisons. 3d Am. Ed. p. 55.

§ Rel. Animal Dis. to Public Health. p. 141.

and a peculiar sensation in the hands and fingers. These mild phenomena continued about five days, then the lining of the mouth became swollen, especially the covering of the tongue; in a short time vesicles began to develop, and at the same time that these eruptions appeared on the mouth and lips, an eruption of like character was observed on the hands and fingers." Two medical friends of Hertwig subjected themselves to like treatment with similar results. Young animals drinking the milk perish from gastro-enteritis, and Bollinger quotes cases of infection to man by the use of buttermilk.* "It is especially from the vesicles on the teats that the poison may escape and mix with the milk, which, if drank warm, induces inflammation of the fauces, irritation of the alimentary canal, and even eruptions of the skin." † Simmonds and Brown have given the disease to pigs and cats by feeding them with the the milk. The milk is harmless when boiled.

COMPOSITION OF MILK IN ECZEMA EPIZOOTICA. ‡

| | |
|---|---|
| Water | 88.1 |
| Fat | 2.9 |
| Casein | 3.4 |
| Lactin | 5.48 |
| Ash | 68 |
| | 100.56 |

*Milk Sickness, Trembles.*—In some portions of the Southern and Western States—notably among the fertile valleys and coves of the mountainous regions of Kentucky, Tennessee, North Carolina and Georgia—the cattle are liable to the above named disorder, and may, through the medium of their milk, communicate it to man or the lower animals. The districts infested by it are seldom more than a few miles in extent, located in valleys or coves, on the north side of a mountain. The sun shines on these but a few hours daily, if at all; the soil is rich and full of moisture, the trees gigantic and interlacing overhead, the atmosphere damp and chill, like that of a

* Billings. Relation of Animal Diseases to Public Health. p. 55.

† John Gamgee. Veterinarian Vade Mecum. p. 216.

‡ Smee, on Milk in Health and Disease. London, 1875. p. 110.

deep cellar. A well-marked symptom in cattle is an incapacity for muscular exertion, and this is so characteristic of the disease that suspected cattle are always sharply driven before slaughter. The disorder lasts from seven to ten days in cattle, and it is stated that they are never attacked if kept off the pestilential districts until the dew has risen. The symptoms and post-mortem appearance in cattle are those of gastro-intestinal inflammation.

In man, the symptoms somewhat resemble those of typhus fever, and the post-mortem appearances are destruction of the epithelium along the digestive tract, with ulceration and suppuration, the gall bladder distended with inky bile, the liver softened, the spleen enlarged, and its contents grumous.*

*Causation.*—It is probably due to the presence of some undiscovered parasite; the fact that the cause disappears with the rising and returns with the falling dew is presumptive of such origin. It has been attributed to feeding on the rhus toxicodendrun, white snake root and eupratorium ageratoides. Hooker has seen a similar disease in New Holland, and attributes it to the cattle eating certain leguminous plants belonging to the genera "gmopholobium."

The poison has an affinity for oil and is removed from the milk to the butter by churning.

*Tuberculosis* (Perlsucht).—Considerable discussion has arisen as to the possibility of the transmission of bovine tuberculosis to man. The fact that it has been transmitted to other animals by means of the milk is strong presumptive evidence that it may be given to man in like manner, and as direct experiment is of course out of the question, we must be satisfied to secure and carefully weigh all the evidence we can accumulate.

The experiments of Gerlach and others prove that it is easily and promptly transmitted to pigs, rabbits and guinea pigs.

Creighton says † "There is little doubt that the pearl disease, modified only in the relative development of the process in various organs, has been communicated to animals by experiment. "There is evidence also that it has been communicated

* Dickson's Elements of Medicines. 2d Ed., p. 488.

† Bovine Tuberculosis in Man. p. 101, *et seq.*

to animals by accident. Has it ever been communicated by accident to man? There is no lack of reasonable presumption that it has. The analogous disease of the horse (glanders) is now and then communicated to man, but we are brought in much closer contact with the bovine than with the equine race. "Besides bread, there is hardly a more universal article of diet than cows' milk. There are many more tuberculous cows than glandered horses, and it would take a good deal to reassure us that we do not sometimes partake of their tainted milk. Such arguments are plausible enough, but they do not prove that bovine tuberculosis ever has been communicated to man. I do not see how such communication can ever be proved, except by the evidence that the form and structure of the morbid products are the same as those of the bovine pearl disease." Under what names then have we been speaking of bovine tuberculosis in the past? First and foremost, there are the sudden and unaccountable onsets of tuberculosis both in children and adults. There is a growing conviction that such cases are due to the introduction of a specific virus into the body, and it is a question of morphological evidence whether in any particular case the morbid products are those that would be due to the specific bovine virus. It is the evidence of identity in form and structure that I have dwelt so much on in this work, and I would claim no case of tuberculosis in man as a case of communicated bovine tuberculosis unless I found on the serous membrane (especially on the sharp margins of the lungs and on the under surface of the diaphraghm), or in the lymphathic glands, or in all these together, and occasionally in other parts or organs, those evidences both of form and minute structure which are distinctive of the bovine disease as it exists primarily and specifically in the cow. The deposits in perlsucht are most commonly found on the serous surfaces in cattle, the mesentery, omentum, peritonium and pleura, often containing many pounds of them. The lungs in many of the cases coming under my notice have been invaded late, the difficulty in respiration being often due to the enormous infiltration of the bronchial lymphatic glands, and not to changes in the lung substance. In one case the axillary glands were affected to such an extent as to produce serious deformity in the region of the scapula humeral articulation. In this case I was sent for to *reduce a dislocation of the shoulder joint*, and the owner was very indignant when told that the animal had tuberculosis. A post-mortem examination in this case showed almost every tissue and organ of the body affected in some degree. Oftentimes the first outward and visible sign of the disorder is mastitis, and the veterinarian must very carefully exclude tuberculosis in making a diagnosis in these cases. I have seen two instances where the consumption of tuberculous milk was followed by symptoms of lung trouble and general wasting. The cow was destroyed in each case (in one she had been procured to furnish milk for a delicate lady and her infant) and the symptoms in one instance were removed. In the other case I lost sight of the family. Physicians attending cases of threatened tuberculosis should always investigate the condition of the cows furnishing the milk supply to the patient, or have a competent veterinarian do it for them.

### DISEASES PECULIAR TO THE HUMAN SPECIES WHICH MAY ME CARRIED IN MILK.

Mr. Ernest Hart has collected and tabulated fifty epidemics of enteric fever, fifteen of scarlet fever and seven of diphtheria which have been traced to milk poisoning.* Where enteric fever is conveyed the medium is usually contaminated with water used to wash the cans or adulterate the milk. Scarlet fever is more likely to be propagated by the desquamating cuticle getting into the milk. Mr. Powers concludes that therefore though a connection between diphtheria and the consumption of milk has not been proven as yet, still it is very likely indeed. His careful investigations into the cause of some local epidemics in North London, exclude any other source from which the people could have been affected.† Greenhow says: ‡ "The contagion of scarlet fever or smallpox may undoubtedly be conveyed from place to place by means of clothing *or other articles* which have been in contact with the sick, but no such instances of the transmission of diphtheria have

* Parkes Hygiene. W. Wood & Co. Vol. 1, p. 285.

† Jacobs on Diphtheria, p. 64.

‡ Greenhow, on Diphtheria. London, 1860, p. 148.

come under my notice." Mackenzie says:* "In many cases of diphtheria which I have seen during the last few years, the drinking water was found to be contaminated with excrementitious matter." "The poison may be received into the system through the water that is drunk or food that is eaten." Smee quotes the case of the orphan asylum at Beddington, where an outbreak of enteric fever was traced to the milk supply.

One morning one of the children brought to the matron a tadpole found in her mug; the dairy was changed and the outbreak at once ceased. (Smee on "Milk in Health and Disease.) It would be easy to multiply instances of this kind, but it would serve no purpose.

*We know these diseases are transmitted through milk*, and it is the duty of the physician to investigate the milk supply in all doubtful cases.

LEGISLATION RESPECTING MILK.

*New York State.*—Prosecutions for the sale of impure milk are conducted under the food adulteration act. The State Board of Health have adopted as the limit 11.5 total solids with 2.5 fat (the English standard). In New York city prosecutions are carried on under the sanitary code of the Board of Health, which prohibits the bringing into, offering for sale, or selling in the city, any milk from which anything has been taken or to which anything has been added. The lactometer, based on a specific gravity of 1029 at 60° F., is used to detect watered milk, but an analysis of skim milk is made, and when the fat falls below 2.5 per cent. skimming is inferred. Four sanitary inspectors have charge of the work, and are authorized to destroy all impure milk.

*Massachusetts*—State law authorizes cities and towns to appoint milk inspectors, but Boston and Cambridge are the only places where inspectors operate. Any milk falling below 13 per cent. total solids is considered by law as impure. Punishment by fine and publication. (The food adulteration act now in force requires only 12 per cent. solids).

*Rhode Island*—State law authorizes appointment of inspectors. Standard 12 per cent. solids, 2.5 per cent. fat. Penalty $20. Providence is the only city enforcing the law.

---

* Mackenzie, on Diphtheria. London, 1879, pp. 11, 16.

*New Jersey*—Law requires 12 per cent. solids, and prohibits the sale of skim milk in cities of the first-class. Where skim milk is sold in other places it must be carried in cans stamped "Skim Milk" in letters two inches high. The penalty for the first offence is $50 ; for the second $100, with forfeiture of the condemned milk. The sale of milk from cows fed on distillery swill or other unwholesome food, or of milk exposed to the emanations from contagious diseases is prohibited. Cincinnati, Cleveland, Syracuse, Paterson and Newark, N. J., have local laws, some of them including inspection of the dairies.

